# Prevalence of pre-frailty and frailty and their association with postoperative hospital days in older adults referred for elective orthopaedic surgery

**DOI:** 10.1007/s41999-026-01515-w

**Published:** 2026-06-08

**Authors:** Marie Linderup Funck, Lotte Sejr Kirring, Anne Mette Schmidt, Inger Markussen Gryet, Morten Fenger-Grøn, Peter Vedsted, Merete Gregersen

**Affiliations:** 1 Medical Diagnostic Center, University Clinic for Innovative Patient Pathways, Regional Hospital Central Jutland, Silkeborg/Viborg, Denmark; 2 University Clinic for Interdisciplinary Orthopaedic Pathways, Elective Surgery Center, Regional Hospital Central Jutland, Silkeborg, Denmark; 3 Department of Geriatrics, Aarhus University Hospital, Aarhus, Denmark; 4 Department of Clinical Medicine, Aarhus University, Aarhus, Denmark

**Keywords:** Frailty, Geriatric assessment, Aged, Orthopaedic surgery

## Abstract

**Purpose:**

The aim of this study was to investigate the prevalence of pre-frailty and frailty among older persons referred for elective orthopaedic surgery and determine whether frailty was associated with the occurrence of postoperative complications, measured as the number of days spent in the hospital within 90 days after surgery.

**Methods:**

Between April 1 and July 31, 2022, we consecutively included individuals aged 65 years or older who were referred for elective shoulder, knee, hip, or spine surgery at a centre for elective surgery. Health status was assessed preoperatively using the record-based Multidimensional Prognostic Index, based on data extracted from the electronic medical record by a geriatrician. Postoperative hospital data were obtained from the Business Intelligence Data Warehouse of the Central Denmark Region.

**Results:**

A total of 519 persons were included, with a mean age of 75.2 years (SD 5.9), and 56% were females. The majority of the persons were referred for elective hip (41%) or knee (37%) arthroplasty. Preoperatively, 26% of the persons were classified as pre-frail and 4% as moderately frail, while none were severely frail. Pre-frailty and moderate frailty were associated with a greater number of hospital days within 90 days post-surgery compared with non-frail persons.

**Conclusion:**

More than one in four older persons aged 65 + referred for elective orthopaedic surgery are pre-frail or moderate frail. Identifying this vulnerability is essential for optimizing care across healthcare settings and professions, with the goal of preventing postoperative complications.

## Key summary points

**Aim:**

To assess the prevalence of pre-frailty and frailty in persons aged 65 and older referred for elective orthopaedic surgery, and to explore its association with postoperative complication risks.

**Findings:**

Our study revealed that 29% of older persons referred for elective orthopaedic surgery were classified as pre-frail or moderate frail, a condition associated with higher complication rates, reflected in longer hospital stays within 90 days post-surgery.

**Message:**

Identifying pre-frailty and frailty in older persons referred for elective orthopaedic surgery is crucial for designing tailored interventions aimed at preventing postoperative complications.

## Introduction

Demographics trends indicate a growing number and proportion of older people in society [1]. Consequently, an increasing number of people are expected to undergo surgery for age-related conditions, such as osteoarthritis of the joints [2]. These surgeries are primarily intended to improve physical function and relieve pain, and they are often highly successful. However, many older adults referred for elective procedures may also have compromised overall health [3]. Frailty, which increases with age and the progressive decline of organ systems, is characterized by diminished physiological reserve and greater vulnerability to stressors such as infections, surgery, and environmental changes [4, 5].

In older adults, recognizing a state of frailty can be difficult without a systematic approach. In geriatric wards, the Comprehensive Geriatric Assessment (CGA) [5, 6] is used as an interdisciplinary clinical tool to identify medical conditions, social challenges, and physical and mental limitations. When followed by appropriate interventions, CGA in various healthcare settings—such as general medical wards, geriatric wards, emergency units, and surgical units—has been shown to reduce postoperative complications, shorten hospital stays, decrease mortality, and enhance physical function [7–12]. Therefore, it is crucial to address and manage frailty in older adults systematically to prevent complications, minimize functional decline, and improve surgical outcomes.

The prevalence of frailty among older adults admitted for elective orthopaedic surgery remains unknown. However, across nine observational studies including general surgical patients with mean age ranging from 61 to 77 years, the prevalence of pre-frailty ranges between 31 and 46%, and frailty prevalence between 10 and 37% [13]. The incidence of postoperative complications is 24% in the subgroup of patients with frailty, 9% in the pre-frail and 5% in the non-frail subgroups [13]. In older patients admitted for elective orthopaedic surgery, the relationship between frailty and adverse postoperative outcomes in this population requires further investigation. Therefore, our objectives were to 1) determine the prevalence of pre-frailty and frailty among patients aged 65 + years, and 2) explore the association between pre-frailty and frailty and the total number of hospital days within 90 days following surgery as a proxy for postoperative complications.

## Methods

The reporting follows the STROBE guidelines [14].

### Design and setting

This study was conducted as a retrospective cohort study.

At the Centre for Elective Surgery at Silkeborg Regional Hospital, patients are referred for elective orthopaedic surgery by their general practitioners. In 2022, the centre conducted a total of 3,260 orthopaedic surgeries [15].

Patients undergo a preoperative evaluation approximately four or more weeks before surgery, conducted by an orthopaedic surgeon and an anaesthesiologist to assess surgical indications and anaesthesia considerations. An orthopaedic nurse collects additional patient information in a structured format, including functional status, social conditions, nutrition, excretory function, and measurements of height and weight. The nurse also ensures that patients fully understand the surgical preparations and postoperative care plan. All information is documented in the patient’s electronic medical record (EMR).

### Study population

Between April 1 and July 31, 2022, all patients aged 65 years or older who underwent elective shoulder, hip, or knee arthroplasty or spine surgery at the Centre for Elective Surgery, Silkeborg Regional Hospital, Central Denmark Region, were consecutively included in the cohort, with only one admission per patient included. This period represented the department’s typical workload.

### Data collection

In this retrospective study, we used the record-based Multidimensional Prognostic Index (rMPI) [16] to evaluate the health status of older adults and to identify pre-frailty and frailty. The rMPI contains the same health domains as the bedside-assessed MPI, which is derived from the CGA [17, 18]. Specifically developed to assess health domains from the patient’s medical records, the rMPI allows for the retrospective identification of frailty status [19]. It incorporates domains such as social conditions, number of medications, comorbidities and their severity (measured by the Cumulative Illness Rating Scale-Geriatrics [20]), Activities of Daily Living (ADL) and Instrumental ADL (measured by the Functional Recovery Score [21]), and nutritional status (measured by the Mini Nutritional Assessment Short-Form [22]). Cognitive functioning (measured by the Short Portable Mental Status Questionnaire [23]) and risk of pressure ulcer (measured by the Exton Smith Scale [24]) are also parts of the CGA, but not recorded in the EMRs and were therefore not included in the rMPI. The total scores of all domains are weighted and aggregated in a total sum score ranging from 0 to 1, which is then categorized into four groups: no frailty (< 0.25), pre-frailty (0.25–0.33) [25], moderate frailty (0.34–0.66), and severe frailty (> 0.66). All the scoring tools included in the rMPI are validated [20, 23, 26].

For each patient, the EMR was thoroughly reviewed up until the date of surgery. The EMR includes health information from doctors, nurses, physiotherapists, and occupational therapists, as well as referrals from primary care and details on age, sex, and civil status. It also contains information from previous hospital encounters, including both unplanned and elective admissions, as well as outpatient visits for chronic conditions. Data collection was carried out by a geriatrician with expertise in the CGA model and the rMPI method, as well as clinical experience in frailty assessment.

Outcome data comprised the total number of hospital days within 90 days after surgery, including both the length of the initial stay and any subsequent hospitalizations. Data were retrieved from the BI Data Warehouse of the Central Denmark Region [15].

In Denmark, each individual is assigned a unique personal identification number, which links all health data to that individual [27]. Data were securely registered and managed using the Research Electronic Data Capture (REDCap) software system [28].

### Statistical analysis

Patient characteristics were presented according to health status as frequencies and means, as appropriate, and compared using the chi-square (ꭓ2) test or Fisher’s exact test for nominal categorical variables and analysis of variance (ANOVA) or the Kruskal–Wallis test for numerical or otherwise ordinal variables, as appropriate. For both length of hospital stays and number of postoperative hospital days within 90 days after surgery, frailty group means and relative group differences were estimated using negative binomial models. Corresponding 95% confidence intervals (CIs) as well as p-values for (linear) trend of frailty group were calculated using robust variance estimation to account for possible model misfit of the highly skewed data. Comparisons of frailty groups were performed in a crude version and under adjustment for age and sex.

All analyses were performed using Stata software, version 18 (Texas, USA), with p-values < 0.05 considered statistically significant.

### Ethical approval

Under Danish legislation and the Act on Biomedical Research Ethics Committees, register-based research does not require approval from an ethics committee (§14, section 2). In line with the guidelines set by the Danish Data Protection Agency, permission for data collection from electronic medical records was granted by the hospital management at Silkeborg Regional Hospital. The study was conducted in accordance with the principles outlined in the Helsinki Declaration [29].

## Results

**Fig. 1:**
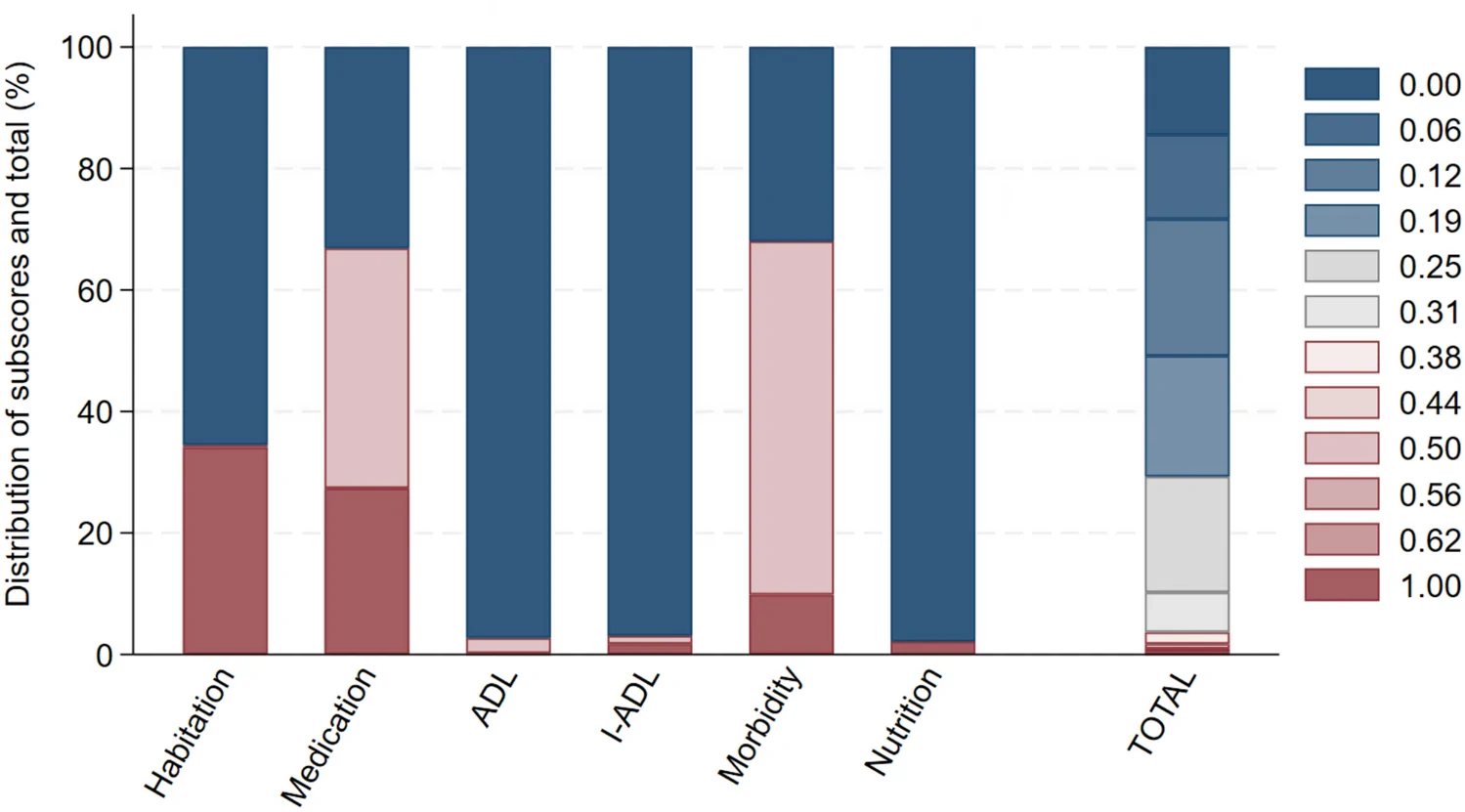
Distribution of health status elements assessed by the Record-based Multidimensional Prognostic Index in older persons referred to elective surgery. Scores are between 0 and 1 (0 = no frailty to 1 = frailty)

A total of 519 consecutive patients were included in this study (Table 1). The mean age of the cohort was 75.2 years (SD 5.9), with men having a mean age of 74.8 years and women 75.5 years (difference = −0.65, 95% CI −1.67 to 0.38). Fifty-six per cent of the patients were females. The majority were referred for elective hip surgery (41%) and knee surgery (37%), while fewer were referred for spine surgery (19%) and shoulder surgery (2%). The median time from referral to surgery was 23 days (interquartile range [IQR]: 12–38). The frequency of pre-frailty was 26%, and moderate frailty was present in 4% of patients. The most frequent contributors to assessed frailty were multimorbidity, polypharmacy, and living alone (Fig. 1). No severely frail patients were referred for elective surgery. One patient without frailty died 53 days after surgery. Compared to non-frail patients, those classified as pre-frail or moderately frail were more often women, older in age, more likely to living alone, and had poorer overall health with impaired physical abilities.

**Table 1:** Baseline characteristics of the 519 patients aged 65 + years referred to elective surgery during the inclusion period measured by the record-based Multidimensional Prognostic Index

	Non-frail *n* = 367	Pre-frail *n* = 133	Moderately frail *n* = 19	*p*-value
Females, *n* (%)	189 (52)	91 (68)	13 (68)	0.002
Mean age, years (SD)	74.5 (5.8)	76.4 (5.7)	79.7 (6.9)	< 0.001
Surgery, *n* (%)				
Knee	144 (39)	45 (34)	4 (21)	0.40
Spine	65 (18)	29 (22)	7 (37)	
Hip	150 (41)	56 (42)	8 (42)	
Shoulder	8 (2)	3 (2)	0 (0)	
Cohabiting status, *n* (%)				
Co-habitant	300 (82)	36 (27)	4 (21)	< 0.001
Institution	0 (0)	0 (0)	2 (11)	
Living alone	67 (18)	97 (73)	13 (68)	
Medication, *n* (%)				
1–3 drugs	169 (46)	3 (2)	0 (0)	< 0.001
4–7	134 (37)	65 (49)	6 (32)	
≥ 8	64 (17)	65 (49)	13 (68)	
ADL^1^-limitation, *n* (%)				
None	367 (100)	127 (95)	11 (58)	< 0.001
Moderate	0 (0)	5 (4)	7 (37)	
Severe	0 (0)	0 (0)	1 (5)	
IADL^2^-limitation, *n* (%)				
None	367 (100)	127 (95)	9 (47)	< 0.001
Moderate	0 (0)	5 (4)	2 (11)	
Severe	0 (0)	1 (1)	8 (42)	
Level of comorbidity, *n* (%)				
Low	155 (42)	10 (8)	1 (5)	< 0.001
Moderate	205 (56)	88 (66)	9 (47)	
Severe	7 (2)	35 (26)	9 (47)	

^1^ *ADL* Activities of Daily Living

^2^ *IADL* Instrumental Activities of Daily Living

*SD* Standard deviation

*IRQ* Interquartile range

Pre-frail and moderately frail patients had longer initial hospital stays than non-frail patients: 2.8 days (95% CI 2.5–3.1) and 3.4 days (95% CI 2.6–4.4), respectively, versus 2.2 days (95% CI 2.0–2.4) among the non-frail patients (Table 2). Within 90 days, 36 patients (7%) experienced at least one readmission, with the highest incidence observed among moderately frail patients (Table 2).

**Table 2:** Mean number of postoperative hospital days within 90 days and relative incidence of hospital days with non-frail patients as reference

Groups	Mean number of postoperative hospital days	Crude variance estimation 95% CI	Adjusted variance estimation 95% CI
Non-frail, *n* = 367	2.2 (2.0;2.4)	Reference	Reference
Pre-frail, *n* = 133	2.8 (2.5;3.0)	0.6 (0.3;0.9)	0.5 (0.2;0.8)
Moderately frail, *n* = 19	3.4 (2.5;4.3)	1.2 (0.2;2.1)	1.0 (0.1;1.9)

	Group means	Relative incidence, crude (95% CI)	Relative incidence, adjusted^a^ (95% CI)	*p*-value for linear trend (adjusted^a^)
Length of initial hospital stay^b^				< 0.001
Non-frail, *n* = 367	2.2 (2.0;2.4)	Reference	Reference	
Pre-frail, *n* = 133	2.8 (2.5;3.1)	1.3 (1.1;1.4)	1.2 (1.1;1.4)	
Moderately frail, *n* = 19	3.4 (2.6;4.4)	1.5 (1.2;2.0)	1.4 (1.1;1.8)	
Number of subsequent hospital days^c^				0.03
Non-frail, *n* = 367	0.5 (0.3;0.9)	Reference	Reference	
Pre-frail, *n* = 133	0.8 (0.3;2.2)	1.7 (0.5;5.6)	1.3 (0.4;4.6)	
Moderately frail, *n* = 19	2.6 (1.1;6.1)	5.7 (2.0;16.1)	6.7 (2.1;21.5)	

*CI* Confidence Interval

*Adjusted for sex and age

^a^Adjusted for sex and age

^b^The day of operation is included as the first day, i.e. minimal length of stay is one day

^c^Number of subsequent hospital days are calculated as (the sum of) discharge date minus admission date, i.e. only overnight stays contribute

## Discussion

In this cohort of patients aged 65 years or older referred for elective orthopaedic surgery, 29% were classified as pre-frail or moderately frail. Preoperative frailty was associated with longer index hospital stays and a higher risk of additional hospital days within 90 days post-surgery, indicating greater postoperative complications. Key factors identified through frailty assessment included higher morbidity, polypharmacy, and living alone, the latter potentially contributing to social isolation.

In comparison to older community-dwelling adults living independently in their own homes, the prevalence of frailty observed in our study is nearly three times higher than the weighted average of 10.7% reported in the systematic review by Collard et al. [3]. Notably, our assessment also included older adults classified as pre-frail, with rMPI scores between 0.25 and 0.33; in the review, pre-frailty was reported in 41.6% of participants across 15 studies [3]. Therefore, it is reasonable to suggest that the cohort of older adults referred for elective orthopaedic surgery represent a distinct population compared with the general community-dwelling older population in Western countries. Nonetheless, in Denmark, orthopaedic surgery is often offered to older adults irrespective of their pre-frailty status.

Since no national or international studies have examined the prevalence of frailty in the older population of elective orthopaedic surgery patients, comparisons can instead be drawn from the occurrence of comorbidities in older adults undergoing hip and knee arthroplasty [30, 31]. Although comorbidity is not a direct measure of frailty, it is a key component of the rMPI, indicating that frail patients are likely also represented in these surgical populations. A prospective observational study with longitudinal patient follow-up would help provide an accurate and nuanced understanding of pre-frailty and frailty in this population.

Upon examination of comorbidity and polypharmacy, it appears that there may be room for improvement through a review of inappropriate medication for older people referred to surgery. Incorporating a structured medication review as part of a geriatric consultation for older adults not admitted to geriatric wards has been shown to enhance the detection of drug-related issues and reduce polypharmacy [32]. Additionally, living alone is associated with an increased risk of acute hospitalization in older adults, particularly among men [33]. Therefore, fostering close collaboration with municipal home care services and engagement of patients’ relatives is essential to prevent re-hospitalization [34].

This study highlights that older adults living with pre-frailty and moderate frailty are at greater risk of postoperative complications, underscoring the importance of incorporating frailty assessments into routine orthopaedic practice. Such assessments would enable proactive, person-centred management strategies to address the increased risks associated with frailty and ultimately improve patient care and outcomes. However, current prehabilitation programmes are largely standardized for non-frail patients, with comorbidities primarily considered in relation to anaesthetic and surgical risk rather than through a holistic care approach. In addition, preoperative information is often delivered digitally, which may exclude patients with limited access to or familiarity with technology. These limitations highlight a gap in specialized prehabilitation for pre-frail and moderately frail patients, warranting future prospective studies into the integration of frailty-informed, person-centred preoperative care pathways.

Preoperative and perioperative strategies may include exercise, nutritional optimization, stabilization of chronic conditions, and coordination with home care services for older patients with limited social support. For cognitively impaired patients, tailored delirium prevention strategies could be planned in advance, as postoperative delirium is a known complication that may result in prolonged hospitalization in older patients [35]. Furthermore, early discharge planning could be initiated to better support preparation for the older patient’s return home [36].

### Strength and limitations

We consecutively included all older adults referred for elective surgery during a defined time period, with complete follow-up, thereby ensuring comprehensive data collection and minimizing the risk of selection bias. However, caution is warranted when interpreting frailty prevalence within the spine and shoulder surgery subgroups due to the small number of cases. Health status was assessed by a single trained and experienced geriatrician using the well-established rMPI tool which has demonstrated strong validity in identifying frailty in both clinical practice and research [19]. As all evaluations were performed by the same clinician, consistency was high and variation minimal. However, we did not conduct inter- or intra-rater variability analyses to further examine potential information bias. Nevertheless, previous studies have shown the rMPI to be valid with minimal variation in clinical settings.

We chose the rMPI tool for health assessment because it is validated for retrospective use, whereas most other frailty scores require bedside evaluation or direct patient assessment. However, the rMPI was originally validated in hospitalized patients, where more comprehensive information from multiple healthcare professionals and a CGA may have been available. In particular, ADL and IADL data may have been difficult to extract reliably from the EMRs, as these measures were not assessed systematically. When certain information was missing in our data collection, we assumed the patient had no limitations, which may have led to an underestimation of frailty prevalence. Consequently, the true prevalence of pre-frailty and frailty in this cohort may have been higher than observed.

All data were extracted from the EMRs prior to surgery, reflecting the standard clinical process for patients awaiting elective procedures. The data collection period corresponded to the department’s typical workload. Notably, postoperative hospital stays have shortened over recent years, with the median length of stay in 2024 being 1 day, leaving limited potential for further reductions. In light of this trend, we estimated the likelihood of patients experiencing hospital stays of five or more days within 90 days after surgery.

We conducted a negative binomial analysis, adjusting for age and sex, as these are potential confounders of prolonged hospital stays. We did not adjust for variables such as social position, which may act as intermediate factors. Our analysis focused exclusively on the number of hospital days and did not include data on medications (e.g. opioids or antibiotics), outpatient visits, or general practice contacts. Accordingly, a composite outcome for complications was used, and more specific outcomes, including mortality, prolonged wound healing, delirium, and infections, were not examined. Therefore, the outcome should be interpreted as a proxy for a potential increase in healthcare needs and complication rate.

### Clinical implications

Older adults are at greater risk of postoperative complications and functional decline compared with younger adults [4, 5]. This study highlights that older adults living with pre-frailty and moderate frailty should be considered at a greater risk of postoperative complications. Our findings emphasize the importance of incorporating frailty assessments into routine clinical practice within orthopaedic surgery, allowing for proactive management strategies to address the increased risks associated with frailty and ultimately improve patient care and outcomes. Further research is needed to explore interventions aimed at addressing the specific needs of pre-frail and frail orthopaedic patients and optimizing their perioperative care. These findings align with the recommendations from the Centre for Perioperative Care and the British Geriatric Society for managing older patients with frailty undergoing surgery in general [37]. Key elements of the recommendations include patient and family involvement, CGA, preoperative risk evaluation, optimization of physical health, proactive discharge planning, and collaboration with primary care providers. Also, identifying older adults living with frailty is essential to ensure they receive targeted geriatric perioperative care interventions and support patient–provider decision-making.

## Conclusion

We found that more than one in four older adults aged 65 or older referred for elective orthopaedic surgery are pre-frail or moderately frail. These conditions were associated with more days spent in hospital post-surgery. The next step is to identify pre-frail and moderate frail older adults to enable the implementation of interdisciplinary preventive interventions.

## Data Availability

The data that support the findings of this study are available from the corresponding author upon reasonable request.
